# Survey based dataset on automation decisions for assembly systems in Germany

**DOI:** 10.1016/j.dib.2020.105782

**Published:** 2020-05-29

**Authors:** Peter Burggräf, Johannes Wagner, Matthias Dannapfel, Sarah Fluchs, Katharina Müller, Benjamin Koke

**Affiliations:** a Chair of International Production Engineering and Management of University of Siegen; b Laboratory for Machine Tools and Production Engineering (WZL) of RWTH Aachen University; c Chair of Production Engineering of E-Mobility Components

**Keywords:** Assembly, Automation, Cost-benefit analysis, decision-making, questionnaire survey

## Abstract

The data provided in the present article provides information on the importance of a list of monetary and non-monetary influencing factors on decisions regarding the automation of an assembly system. A survey among German industry representatives conducted between July 2018 and October 2018 is the basis for this dataset. It contains the characteristics of industrial companies based in Germany that participated in the survey as well as their attitude towards the development of the automation level in assembly systems. The focus of the survey lies on the influencing factors of the production and production environment and their influence on the automation level. The participants were able to evaluate the factors on a six-step ordinal scale from “no influence” to “very strong influence”. Interpretation of this data can be found in the research article titled “Automation decisions in flow-line assembly systems based on a cost-benefit analysis” [1].

Specifications table

**Table:** 

Subject	Engineering
Specific subject area	Assembly systems in production
Type of data	Table
How data were acquired	Online Survey
Data format	Raw
Parameters for data collection	Targeted participants were industry representatives in German speaking countries. Participation was voluntary and without compensation. The sample was addressed through mass mailing to known interest groups.
Description of data collection	Data were collected through an online survey as a self-administered questionnaire developed within a research project.
Data source location	Institution: University of Siegen City/Town/Region: Siegen Country: Germany
Data accessibility	Mendeley Data https://data.mendeley.com/datasets/brb9jb929y/1
Related research article	[1] Burggräf, P., Wagner, J., Dannapfel, M., Fluchs, S., Müller, K., & Koke, B. (2019). Automation decisions in flow-line assembly systems based on a cost-benefit analysis. Procedia CIRP, 81, pp. 529-534.

## Value of the Data

• The dataset is valuable as it records information on the anticipated development of the automation level in German manufacturing companies in the area of assembly.
• The dataset contains monetary factors as well as non-monetary factors, which are to be included in the decision making process regarding the automation level in assembly systems.
• The dataset can contribute to further research on potential strategies for the development of automation in assembly systems.
• The dataset can be used as reference value to compare the view of German companies with those of other countries.

## 1 Data Description

The *supplemental data* provided in this article contains three data files relevant to the conducted online survey.

The *pdf*-File “Appendix A Questionnaire” shows a printout of the survey design as it has been presented to the survey participants.

The *pdf*-File “Appendix B Variables” provides an overview of the variables reported by the system. This includes questionnaire-internal data as provided by the questionnaire system SosciSurvey, a description of every question plus the corresponding category, and the answer texts mapped to the variables shown in the raw data.

The *MS Excel*-file “Data Set” contains the raw data of questionnaire responses. The dataset shows system-generated values such as case number, Serial number, reference, questionnaire name, mode, start time and various time flags per page, as well as responses to 87 questions among which 12 were of demographic nature. Furthermore, the final columns hold analytic data provided by the system, e.g. a binary value for the state “finished”, the last page viewed, the maximum page number viewed, percentages for missing answers, and penalties for fast filling in.

## 2 Experimental Design, Materials, and Methods

The data set linked to aforementioned journal publication contains the participants’ responses from a questionnaire based online survey. It aimed at capturing the responses from industry representatives of different branches, which are involved in assembly planning, assembly operation and automation decisions, on the importance of multiple criteria for automation decisions, focusing especially on the difference between monetary and non-monetary influencing factors.

A structured and pre-tested questionnaire was developed to capture responses amongst industry representatives. In the course of pretests, six test-users received the online test-link and evaluated understandability, effort and structure of the questionnaire. Furthermore, a technical function test was conducted by answering the questionnaire three times and comparing the expected data output with the actual output to ensure a correct coding of the answers in the data set. The questionnaire is presented in Appendix A.

The questionnaire itself is divided into three main sections – Section A “motivational hypotheses”, section B “influencing factors” and section C “demographic questions”.

Section A collects the appraisal on the expected development of automation within the next five years, the perceived importance of the right automation level in assembly systems and the comparison of the importance and practical consideration of monetary and non-monetary factors.

Section B forms the main part of the questionnaire and captures the participants’ opinion on the influence of monetary and non-monetary factors on automation decisions. The list of 19 monetary and 52 non-monetary influencing factors was identified through an extensive literature review [2], [3], [4], [5], [6]. To improve ergonomics for respondents (i.e. avoiding tiresome scrolling through the questionnaire), the factors are presented in sections. On a first level, they are divided into the three views “market”, “technology” and “monetary” and on a second level into areas. The market view contains the areas “market & competitors”, “own company”, “employees” and “customers”. The technology view comprises the areas “technology development & production process”, “product” and “design”. The monetary view lists the monetary factors and is not further broken down.

The data were captured by using a six-point Likert-scale with labeled extremes ranging from “no influence“ to “very strong influence”. In the output data table the rating is expressed by values from 1 to 6. The full key to variables and questions is presented in Appendix B. Furthermore the option “I cannot tell” was given. The aim was to prohibit the possibility to take a neutral position but to indicate a tendency in one direction or deliberately refuse the statement.

Section C captures demographic questions about the characteristics of the companies such as branch or annual turnover and questions about the assembly system of this company such as the current automation level of the assembly system, product type and structure as well as the assembly quantity and personal. Finally, age and gender of the participant are asked for. To assess the current automation level, the seven-point reference scale of the mechanical Level of Automation (LoA) according to the DYNAMO research project is used [7]. Therefore the scale is shown to the survey participance next to the question.

The survey was published as an online questionnaire via the website www.soscisurvey.de, a German survey platform following German data security law. It was online available between July and October 2018. A grand total of 47 questionnaires was returned of which 33 had been completed. mmc1.docx mmc2.docx

## Declaration of Competing Interest

The authors declare that they have no known competing financial interests or personal relationships which have, or could be perceived to have, influenced the work reported in this article.

## Appendix Supplementary materials

Image, application 1

Image, application 1
