# Supplementary material for: Survey based dataset on automation decisions for assembly systems in Germany
Source: Data Brief. 2020 May 29;31:105782. doi: 10.1016/j.dib.2020.105782 (PMC7284056; doi:10.1016/j.dib.2020.105782)
Supplement: Supplementary file 1 [file mmc1.docx]

MAproFli → MAproFLi02

03.11.2018, 12:09

## Page 01


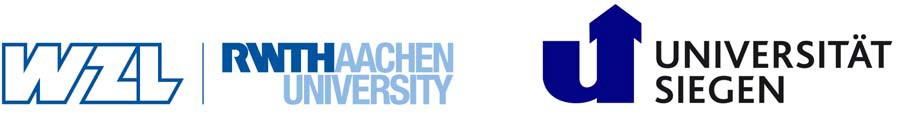


Dear participants,

in the context of a research project of the German Research Foundation (DFG), the Chair of International Production Engineering and Management (IPEM) of University of Siegen and the Laboratory for Machine Tools and Production Engineering (WZL) of RWTH Aachen University are conducting a study on the topic of criteria for automation decisions.

The aim of the research project is to develop a decision methodology for determining the optimal degree of automation in flow line assembly. This study focuses on the importance of non-monetary factors for automation decisions. In this context, the study serves to review and weigh the influencing factors that were developed in the project. Furthermore, a possible dependence of the factors from industry and company size will be investigated.

Altogether, the survey does not take longer than 15 minutes of your time. Please take your time to read through each point and answer as honestly as possible. Each completed questionnaire is a valuable contribution to the survey and contributes to the quality of the results.

Your participation is voluntary and your answers will be treated anonymously. The publication of the results obtained in the context of this study will also be made anonymous.

If you have any questions or comments about this survey or the project, please contact us by e-mail:

K.Mueller@wzl.rwth-aachen.de
Benjamin.Koke@uni-siegen.de

By clicking on "Continue", you indicate that you have read this cover page and that you are voluntarily participating in this survey. You also agree that the anonymized data may be used jointly by the above-mentioned research institutions.

**We thank you for your participation!**

## Page 02

**HT**

First, we ask you to answer some general questions on the subject of automation in assembly. Please select the answer you think is most appropriate.

| **In the next five years the degree of automation will ...** |
| --- |
| 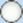 increase.  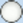 not change.  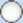 decrease. |

| **In your opinion, what influence does the optimal degree of automation in assembly have on the success of the company?** |
| --- |
| 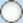 None.  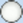 Low.  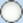 High.  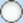 Very high. |

| **Assess the importance of monetary and non-monetary decision factors on the degree of automation. 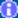** | | | | |
| --- | --- | --- | --- | --- |
| monetary factors much more important | monetary factors  more important | equally important | non-monetary factors  more important | non-monetary factors much more important |
| 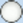 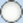 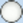 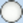 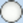 | | | | |
|  | | | | |

| **Which decision factors do you use in your company for automation decisions?** | | | | |
| --- | --- | --- | --- | --- |
| only monetary factors | mostly monetary factors | monetary and non‐ monetary factors in in equal measure | mostly non-monetary factors | only non-monetary factors |
| 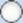 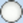 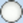 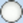 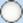 | | | | |
|  | | | | |

## Page 03

**MS**

#
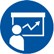
Influencing factors from the market perspective

| **Subcategory: Market and competitors**  Assess the influence of the following factors on the degree of automation of the assembly. Influence means that in theory changes in the influencing factor require an adjustment of the degree of automation. | |
| --- | --- |
| no very strong  influence influence | I  cannot  say |
| Dynamics, laws & development of the market 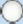 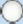 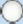 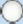 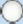 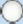 | 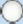 |
| Structure & segmentation of the market 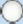 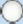 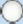 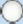 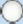 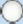 | 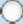 |
| Competitor structure 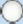 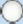 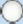 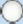 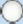 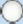 | 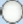 |
| Entry of new competitors 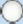 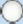 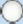 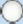 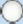 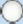 | 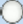 |
| Automation strategy of competitors 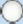 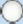 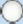 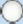 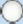 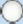 | 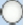 |

##
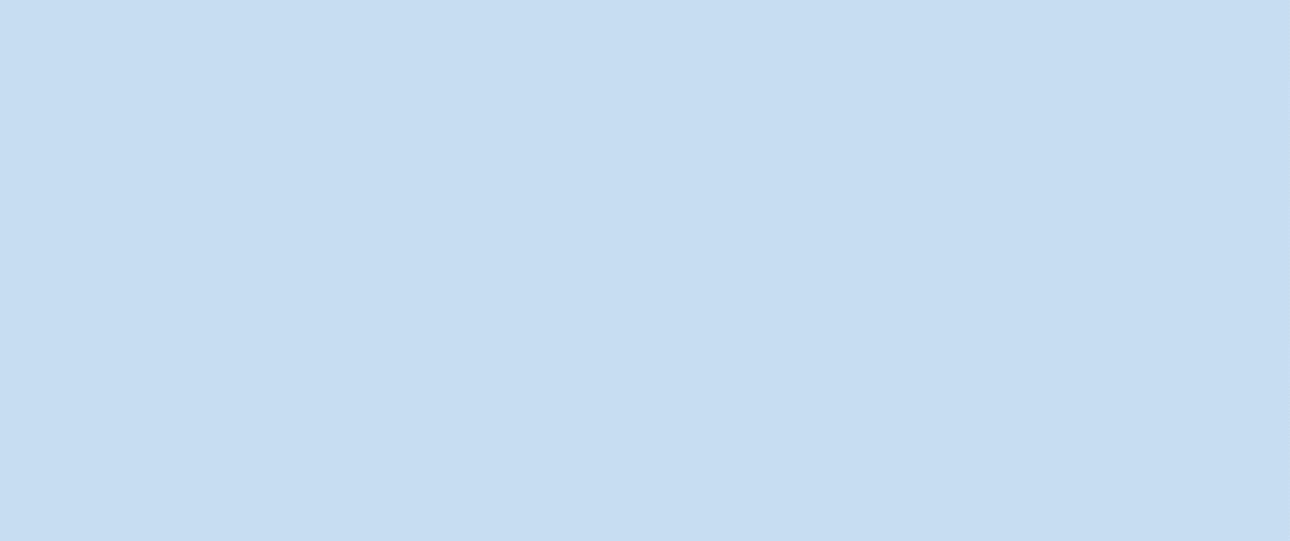
 Page 04

**MS2**

#
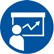
Influencing factors from the market perspective


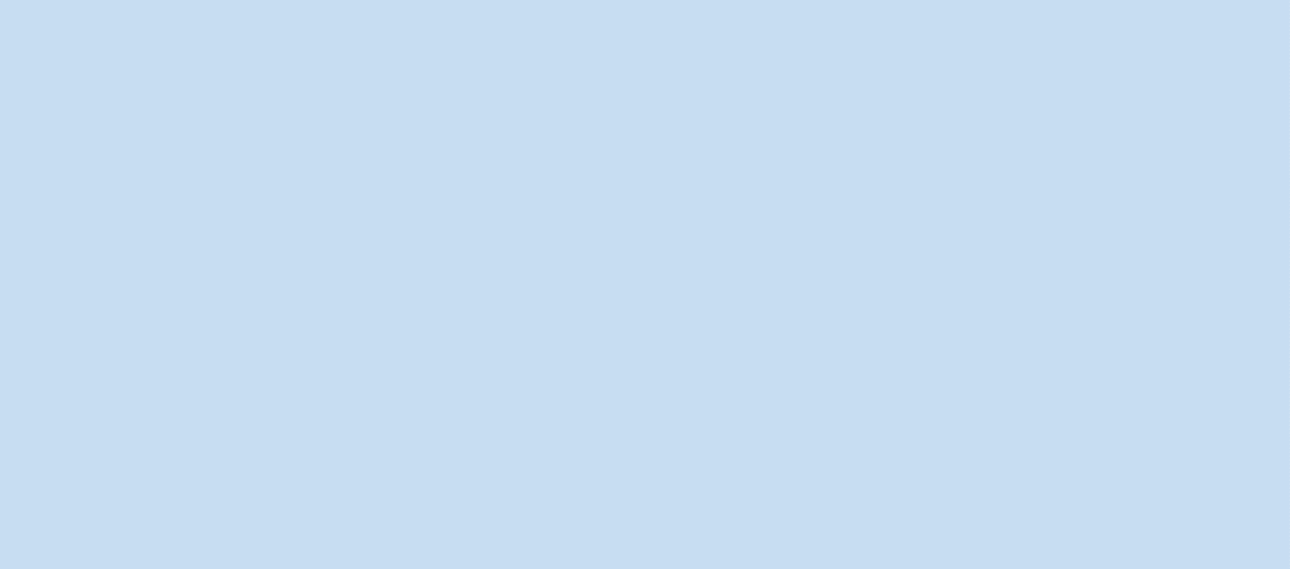


| **Subcategory: Own company**  Assess the influence of the following factors on the degree of automation of the assembly. Influence means that in theory changes in the influencing factor require an adjustment of the degree of automation. | |
| --- | --- |
| no very strong  influence influence | I  cannot  say |
| Core competencies of the company 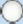 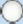 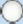 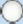 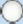 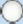 | 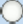 |
| Degree of specialization 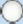 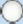 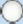 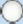 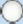 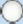 | 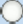 |
| Place of production 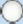 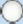 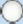 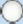 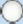 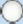 | 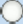 |
| Company size 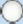 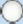 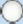 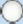 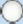 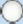 | 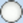 |
| Corporate culture 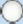 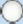 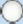 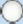 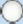 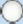 | 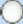 |
| Willingness to invest 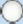 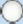 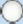 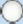 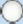 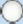 | 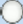 |

## Page 05

**MS3**

# Influencing factors from the market perspective

| **Subcategory: Personnel**  Assess the influence of the following factors on the degree of automation of the assembly. Influence means that in theory changes in the influencing factor require an adjustment of the degree of automation. | |
| --- | --- |
| no very strong  influence influence | I  cannot  say |
| Employee structure |  |
| Qualification of employees |  |
| Qualification measures |  |
| Willingness to change |  |
| Works council and works agreements |  |

## Page 06

**MS4**

# Influencing factors from the market perspective

| **Subcategory: Customers**  Assess the influence of the following factors on the degree of automation of the assembly. Influence means that in theory changes in the influencing factor require an adjustment of the degree of automation. | |
| --- | --- |
| no very strong  influence influence | I  cannot  say |
| Demand development / fluctuations in demand |  |
| Quality requirements |  |
| Delivery requirements |  |
| Price requirements |  |
| Individuality requirements |  |

## Page 07

**TS**

# Influencing factors from the technology perspective

| **Subcategory: Technology development & production process**  Assess the influence of the following factors on the degree of automation of the assembly. Influence means that in theory changes in the influencing factor require an adjustment of the degree of automation. | |
| --- | --- |
| no very strong  influence influence | I  cannot  say |
| Technology level |  |
| Speed of innovation |  |
| Technical standards |  |
| Research and development intensity |  |
| Reusability of the equipment |  |
| Assembly technology |  |
| Availability of information |  |

## Page 08

**TS2**

# Influencing factors from the technology perspective

| **Subcategory: Product**  Assess the influence of the following factors on the degree of automation of the assembly. Influence means that in theory changes in the influencing factor require an adjustment of the degree of automation. | |
| --- | --- |
| no very strong  influence influence | I  cannot  say |
| Duration of the product life cycle |  |
| Product life cycle stage |  |
| Number of product variants |  |
| Product types |  |
| New product launch |  |
| Product quantities/quantities |  |
| Product weight |  |
| Product size |  |
| Product complexity |  |
| Frequency of design changes |  |

## Page 09

**TS3**

# Influencing factors from the technology perspective

| **Subcategory: Construction**  Assess the influence of the following factors on the degree of automation of the assembly. Influence means that in theory changes in the influencing factor require an adjustment of the degree of automation. | | |
| --- | --- | --- |
| no very strong  influence influence | | I  cannot  say |
| Form stability of the joining component | |  |
| Sensitivity of the joining component | |  |
| Gripping surfaces for automated handling on the joining component |  |  |
| Variants of the joining component | |  |
| Enveloping volume of the joining component  (length, width, height) | |  |
| Number of stable component positions | |  |
| Symmetry of the joining component | |  |
| Hooking, jamming, sticking etc. of the joining  component(s) |  |  |
| Defective joining components, foreign parts,  contamination | |  |
| Accessibility of the positioning range | |  |
| Orientation of the joining component before joining | |  |
| Joining movement | |  |
| Joining force or joining moment | |  |
| Joining aid available on joining and base component | |  |

## Page 10

**MF**

# Influencing factors from the monetary perspective

| Assess the influence of the following factors on the degree of automation of the assembly. Influence means that in theory changes in the influencing factor require an adjustment of the degree of automation. | |
| --- | --- |
| no very strong  influence influence | I cannot  say |
| Company turnover |  |
| Company profit |  |
| Financial situation |  |
| Personnel costs |  |
| Production overhead cost |  |
| Development and construction costs |  |
| Machine hour rate |  |
| Depreciation |  |
| Investment budget |  |
| Working time models and remuneration models |  |
| Assembly costs/piece |  |
| Annual output |  |
| Annual assembly costs |  |
| Amortization period for plant and equipment |  |
| Reacquisition value of plant and equipment |  |
| Calculative interest |  |
| Maintenance costs |  |
| Operating time per day in x shifts |  |
| Working days per year |  |

## Page 11

**DF**

| **To which industry does your company belong?** |
| --- |
| Mechanical engineering and plant engineering  Automotive industry  Metalworking  Electronics and electrical engineering  Other |

| **What was the annual turnover of your company in 2017?** | | | | | | |
| --- | --- | --- | --- | --- | --- | --- |
| 0‐10 mil. € | 10‐50 mil. € | 50‐100 mil. € | 100‐250 mil. € | 250‐500 mil. € | 500‐1.000 mil. € | >1 bil. € |
|  | | | | | | |
|  | | | | | | |

| **In your opinion, what degree of automation do you have in the assembly?**   \| **Level** \| **Description** \| **Example** \| \| --- \| --- \| --- \| \| 1 \| Manual only \| Muscle power only \| \| 2 \| Static hand tool \| Screwdriver \| \| 3 \| Flexible hand tool \| Adjustable Screwdriver \| \| 4 \| Automatic hand tool \| Hydraulic riveting tool \| \| 5 \| Static work station \| Lathe machine \| \| 6 \| Flexible work station \| CNC processing center \| \| 7 \| Exclusively automatic \| Autonomous systems \| | | | | | | |
| --- | --- | --- | --- | --- | --- | --- | --- | --- | --- | --- | --- | --- | --- | --- | --- | --- | --- | --- | --- | --- | --- | --- | --- | --- | --- | --- | --- | --- | --- | --- |
| 1 | 2 | 3 | 4 | 5 | 6 | 7 |

| **In your opinion, does this correspond to the optimum degree of automation?** |
| --- |
| It is in the optimal  It is too low. area. It is too high. |

| **To which of the following types do you assign the products of your company?** | | | | |
| --- | --- | --- | --- | --- |
| Standardized products | Predominantly  standardized  products | Predominantly  customer-individual products | Customer-individual products | Different type |
|  | | | |  |
|  | | | | |
| When choosing „Different type“ please explain | | | | |

| **How high were the assembly quantities of your company in 2017 approximately?** | | | | | | | |
| --- | --- | --- | --- | --- | --- | --- | --- |
| up to 50 | up to 100 | up to 1.000 | up to 10.000 | up to 50.000 | up to 500.000 | up to 1.000.000 | over 1.000.000 |
|  | | | | | | | |
|  | | | | | | | |

| **How many parts do your products approximately consist of on average?** | | | |
| --- | --- | --- | --- |
| <100 | 101‐1.000 | 1.001‐10.000 | >10.000 |
|  | | | |
|  | | | |

| **How many employees work in your company in the field of assembly?** | | | | | | |
| --- | --- | --- | --- | --- | --- | --- |
| up to 50 | up to 100 | up to 500 | up to 1.000 | up to 2.500 | up to 5.000 | over 5.000 |
|  | | | | | | |
|  | | | | | | |

## Page 12

| **To what age group do they belong?** | | | | |
| --- | --- | --- | --- | --- |
| 29 or younger | 30‐39 | 40‐49 | 50‐59 | 60 or older |
|  | | | | |
|  | | | | |

| **Please enter your gender.** |
| --- |
| female male not specified |

**Last page**

Thank you for your participation!

We would like to thank you very much for your participation.

If you have any questions or comments about this survey or the project, please contact us by e-mail:

Katharina Müller

Benjamin Koke

Your answers have been saved, you can now close the browser window.

Katharina Müller M.Sc., Laboratory for Machine Tools and Production Engineering (WZL) RWTH Aachen University
Benjamin Koke M.Sc. M.Sc., IPEM, University of Siegen
