# Supplementary material for: Survey based dataset on automation decisions for assembly systems in Germany
Source: Data Brief. 2020 May 29;31:105782. doi: 10.1016/j.dib.2020.105782 (PMC7284056; doi:10.1016/j.dib.2020.105782)
Supplement: Supplementary file 2 [file mmc2.docx]

Overview of variables

**[DF03]** Scale (intermediate values labelled)

Assembly quantity

"How high were the assembly quantities of your company in 2017 approximately?"

**[DF02]** Scale (intermediate values labelled)

Degree of automation

"In your opinion, what degree of automation do you have in the assembly?"

# Questionnaire-internal data

In the data set you will find the following additional variables in addition to your questions, unless you disable the corresponding option when downloading the data set.

**CASE** Consecutive number of the test person

**REF** Refer Reference, if such a reference was provided in the link to the questionnaire

**LASTPAGE** Number of the page in the questionnaire that was last edited and sent

**QUESTNNR** Identification of the questionnaire that was processed

**MODE** Information whether the questionnaire was started in the pretest or by a project member

**STARTED** Time at which the participant called up the questionnaire
**FINISHED** Information whether the questionnaire was completed to the last page **TIME_001...** Time a participant spent on a questionnaire page

Please note that you cannot read the questionnaire internal variables with the value() function. But for interview number and reference the PHP functions
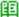
 PHP-Function caseNumber() and
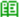
 PHP-Function reference() are available.

Details about the additional variables can be found in the manual:
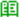
 Additional variables in the data output

**Category DF: Demographic issues**

**[DF01]** Scale (intermediate values labelled)

Position of the products

"To which of the following types do you assign the products of your company?"

**DF01_01** [No description] 01

1 = Standardized products

2 = Predominantly standardized products

3 = Predominantly customer-individual products
4 = Customer-individual products

-1 = Different type

-9 = not answered

**DF02_01** [No description] 01

1 = 1

2 = 2

3 = 3

4 = 4

5 = 5

6 = 6

7 = 7

-9 = not answered

**DF03_01** [No description] 01

1 = up to 50

2 = up to 100

3 = up to 1.000

4 = up to 10.000

5 = up to 50.000

6 = up to 500.000

7 = up to 1.000.000

8 = over 1.000.000

-9 = not answered

**[DF09]** Open text input

Free text for DF01

**[DF08]** Scale (intermediate values labelled)

Opinion on the degree of automation

"In your opinion, does this correspond to the optimum degree of automation?"

**[DF07]** Scale (intermediate values labelled)

Turnover

"What was the annual turnover of your company in 2017?"

**[DF06]** Selection

Industry

"To which industry does your company belong?"

**[DF05]** Scale (intermediate values labelled)

Assembly workers

"How many employees work in your company in the field of assembly?"

**[DF04]** Scale (intermediate values labelled)

Number of components

"How many parts do your products approximately consist of on average? *"

**DF04_01** [No description] 01

1 = &lt;100

2 = 101-1.000

3 = 1.001-10.000

4 = &gt;10.000

-9 = not answered

**DF05_01** [No description] 01

1 = up to 50

2 = up to 100

3 = up to 500

4 = up to 1.000

5 = up to 2.500

6 = up to 5.000

7 = over 5.000

-9 = not answered

**DF06** Branche

1 = Mechanical engineering and plant engineering
2 = Automotive industry

3 = Metalworking

4 = Electronics and electrical engineering
5 = Other

-9 = not answered

**DF06_05** Other

Open text input

**DF07_01** [No description] 01

1 = 0-10 mil. €

2 = 10-50 mil. €

3 = 50-100 mil. €

4 = 100-250 mil. €

5 = 250-500 mil. €

6 = 500-1.000 mil. €

7 = &gt;1 bil. €

-9 = not answered

**DF08_01** [No description] 01

1 = It is too low.

2 = It is in the optimal area.
3 = It is too high.

-9 = not answered

**DF09_01** When choosing „Different type“ please explain

Open text input

**[MH04]** Scale (intermediate values labelled)

Importance

"Assess the importance of monetary and non-monetary decision factors on the degree of automation."

**[MH03]** Selection

Influence

"In your opinion, what influence does the optimal degree of automation in assembly have on the success of the company?”

**[MH02]** Selection

Value

"In your opinion, how important is automation in assembly planning?"

**[DF10]** Scale (intermediate values labelled)

Age

" To what age group do they belong?"

**DF10_01** [No description] 01

1 = 29 or younger

2 = 30-39

3 = 40-49

4 = 50-59

5 = 60 or older

-9 = not answered

**DF11_01** [No description] 01

1 = female

2 = male

3 = not specified

-9 = not answered

**Category MH: Motivation hypothesis**

**[MH01]** Selection

Assembly 5 years

"In the next five years the degree of automation will ..."

**[DF11]** Scale (intermediate values labelled)

Gender

"Please enter your gender."

**MH01** Assembly 5 years

1 = increase.

2 = not change.

3 = decrease.

-9 = not answered

**MH02** Stellenwert

1 = None.

2 = Low.

3 = High.

4 = Very high.

-9 = not answered

**MH03** Einfluss

1 = None.

2 = Low.

3 = High.

4 = Very high.

-9 = not answered

**MH04_01** [No description] 01

1 = monetary factors much more important 2 = monetary factors more important

3 = equally important

4 = non-monetary factors more important

5 = non-monetary factors much more important

-9 = not answered

**[MH05]** Scale (intermediate values labelled)

Usage

"Which decision factors do you use in your company for automation decisions?"

**MH05_01** [No description] 01
1 = only monetary factors
2 = mostly monetary factors

3 = monetary and non-monetary factors in equal measure

4 = mostly non -monetary factors
5 = only non -monetary factors

-9 = not answered

**Category MS: Market perspective**

**[MS01]** Scale (labeled extremes)

## Market overall

**MS01_01** Dynamics, laws & development of the market *
**MS01_02** Structure & segmentation of the market *
**MS01_03** Demand development / fluctuations in demand *
**MS01_05** Quality requirements *

**MS01_06** Delivery requirements *
**MS01_07** Price requirements
**MS01_08** Individuality requirements *
**MS01_09** Competitor structure *
**MS01_10** Entry of new competitors *

## **MS01_11** Automation strategy of competitors * **MS01_12** Core competencies of the company * **MS01_13** Degree of specialization *

**MS01_14** Place of production *
**MS01_15** Company size
**MS01_16** Corporate culture *
**MS01_17** Willingness to invest *
**MS01_18** Qualification measures *
**MS01_19** Workforce potentials *
**MS01_20** Employee structure *
**MS01_21** Willingness to change *

## **MS01_22** Works council and works agreements *

1 = no influence

6 = very strong influence

-1 = I cannot say

-9 = not answered

**[MS02]** Scale (labeled extremes)

Market and competitors "Subcategory: Market and competitors "

**MS02_01** Dynamics, laws & development of the market *
**MS02_02** Structure & segmentation of the market *
**MS02_09** Competitor structure *

**MS02_10** Entry of new competitors *

**MS02_11** Automation strategy of competitors *

1 = no influence

6 = very strong influence

-1 = I cannot say

-9 = not answered

**[MS05]** Scale (labeled extremes)

Customers

"Subcategory: Customers"

**[MS04]** Scale (labeled extremes)

Personnel

"Subcategory: Personnel"

**[MS03]** Scale (labeled extremes)

Own company

"Subcategory: Own company"

**MS03_12** Core competencies of the company *

**MS03_13** Degree of specialization * **MS03_14** Place of production * **MS03_15** Company size
**MS03_16** Corporate culture *
**MS03_17** Willingness to invest *

1 = no influence

6 = very strong influence

-1 = I cannot say

-9 = not answered

**MS04_20** Employee structure *
**MS04_19** Qualification of employees *
**MS04_18** Qualification measures *
**MS04_21** Willingness to change *

**MS04_22** Works council and works agreements *

1 = no influence

6 = very strong influence

-1 = I cannot say

-9 = not answered

**MS05_03** Demand development / fluctuations in demand *

**MS05_05** Quality requirements **MS05_06** Delivery requirements * **MS05_07** Price requirements *
**MS05_08** Individuality requirements *

1 = no influence

6 = very strong influence

-1 = I cannot say

-9 = not answered

# Category TS: Technology perspective

**[TS01]** Scale (labeled extremes)

## Technology overall

**TS01_01** Technology level *

**TS01_02** Speed of innovation *

**TS01_03** Technical standards *

## **TS01_04** Research and development intensity * **TS01_05** Duration of the product life cycle **TS01_06** Product life cycle stage * **TS01_07** Number of product variants

**TS01_08** Product types *

**TS01_09** New product launch *
**TS01_10** Product quantities/quantities *
**TS01_11** Product weight *

**TS01_12** Product size *

**TS01_13** Product complexity *

**TS01_14** Frequency of design changes
**TS01_15** Reusability of the equipment
**TS01_16** Assembly technology *

**TS01_17** Time-to-market/ Time-to-product
**TS01_18** Availability of information *
**TS01_19** Form stability of the joining component
**TS01_20** Sensitivity of the joining component *

## **TS01_21** Gripping surfaces for automated handling on the joining component *

**TS01_22** Variants of the joining component *

## **TS01_23** Enveloping volume of the joining component (length, width, height) *

**TS01_24** Number of stable component positions *

**TS01_25** Symmetry of the joining component *

## **TS01_26** Hooking, jamming, sticking etc. of the joining component(s) * **TS01_27** Defective joining components, foreign parts, contamination * **TS01_28** Accessibility of the positioning range *

**TS01_29** Orientation of the joining component before joining *

**TS01_30** Joining movement *

**TS01_31** Joining force or joining moment

## **TS01_32** Joining aid available on joining and base component *

1 = no influence

6 = very strong influence

-1 = I cannot say

-9 = not answered

**[TS02]** Scale (labeled extremes)

Technology development and production process

"Subcategory: Technology development and production process"

**[TS03]** Scale (labeled extremes)

Product

"Subcategory: Product"

**[TS04]** Scale (labeled extremes)

Construction

"Subcategory: Construction"

**TS04_19** Form stability of the joining component

**TS04_20** Sensitivity of the joining component *

**TS04_21** Gripping surfaces for automated handling on the joining component *

**TS04_22** Variants of the joining component *

**TS04_23** Enveloping volume of the joining component (length, width, height) *

**TS04_24** Number of stable component positions *

**TS04_25** Symmetry of the joining component *

**TS04_26** Hooking, jamming, sticking etc. of the joining component(s) *
**TS04_27** Defective joining components, foreign parts, contamination *
**TS04_28** Accessibility of the positioning range *

**TS04_29** Orientation of the joining component before joining *

**TS04_30** Joining movement *

**TS04_31** Joining force or joining moment

**TS04_32** Joining aid available on joining and base component *

1 = no influence

6 = very strong influence

-1 = I cannot say

-9 = not answered

**TS03_05** Duration of the product life cycle **TS03_06** Product life cycle stage *
**TS03_07** Number of product variants

**TS03_08** Product types *

**TS03_09** New product launch *
**TS03_10** Product quantities/quantities * **TS03_11** Product weight *

**TS03_12** Product size *

**TS03_13** Product complexity *

**TS03_14** Frequency of design changes

1 = no influence

6 = very strong influence

-1 = I cannot say

-9 = not answered

**TS02_01** Technology level *

**TS02_02** Speed of innovation *

**TS02_03** Technical standards *

**TS02_04** Research and development intensity *
**TS02_15** Reusability of the equipment
**TS02_16** Assembly technology *

**TS02_18** Availability of information *

1 = no influence

6 = very strong influence

-1 = I cannot say

-9 = not answered

# Category MF: Monetary factors

**[MF01]** Scale (labeled extremes)

## Monetary factors

**MF01_01** Company turnover **MF01_02** Company profit **MF01_03** Financial situation * **MF01_04** Personnel costs

**MF01_05** Production overhead cost

## **MF01_06** Development and construction costs *

**MF01_07** Machine hour rate **MF01_08** Depreciation *
**MF01_09** Investment budget *

## **MF01_10** Working time models and remuneration models *

**MF01_11** Assembly costs/piece **MF01_12** Annual output * **MF01_13** Annual assembly costs

## **MF01_14** Amortization period for plant and equipment **MF01_15** Reacquisition value of plant and equipment **MF01_16** Calculative interest *

**MF01_17** Maintenance costs

## **MF01_18** Operating time per day in x shifts *

**MF01_19** Working days per year

1 = no influence

6 = very strong influence

-1 = I cannot say

-9 = not answered

# Category EL: Introduction
